# Supplementary material for: Associations between the Home Physical Environment and Children’s Home-Based Physical Activity and Sitting
Source: Int J Environ Res Public Health. 2019 Oct 29;16(21):4178. doi: 10.3390/ijerph16214178 (PMC6862192; doi:10.3390/ijerph16214178)
Supplement: Supplementary File 1 [file ijerph-16-04178-s001.pdf]

Description of Independent variables		
Audit variables	***Calculation***	***Items***
**Physical activity (PA) equipment accessibility and availability summary score**	Each PA item was multiplied by its accessibility rating (A=1, B=2, C=3, D=4). The PA equipment values for the home were then summed.	**Sports equipment**<br>1. Balls (e.g., football, rugby, basketball)<br>2. Bats/Racquets (e.g., cricket, softball, tennis)<br>3. Frisbee<br>4. Skipping rope<br>5. Hula hoop<br>**Transportation equipment**<br>6. Bicycle<br>7. Scooter/skateboard/ripstick/skates<br>**Fitness equipment**<br>8. Stationary (aerobic) exercise equipment (e.g., treadmill, exercise bike, punch bag)<br>9. Weights/toning equipment<br>**Outdoor play equipment**<br>10. Basketball ring<br>11. Fixed play structure (e.g., swings, slide, climbing, sandpit)<br>12. Cubby/Tree house<br>13. Trampoline<br>14. Pool (in ground or above)<br>15. Football goal net<br>16. Swing ball<br>17. Badminton/Volleyball net<br>**Indoor play equipment**<br>18. Pool/snooker table<br>19. Table tennis table<br>20. Table football
**Musical instrument accessibility and availability summary score**	Each musical instrument item was multiplied by its accessibility rating (A=1, B=2, C=3, D=4). The musical instrument values for the home were then summed.	21. Piano/ keyboard<br>22. Drums<br>23. Other instruments (e.g., guitar, trumpet, violin, flute)
**Overall media equipment accessibility and availability summary score**	Each media equipment item in the home was multiplied by its accessibility rating (A=1, B=2, C=3, D=4). The media equipment values for the home were then summed.	**Fixed**<br>24. Television<br>25. VCR/DVD/Blue-ray player<br>26. Pay TV (e.g., Sky)<br>27. TV on demand (e.g., Apple TV)<br>28. Desktop computer<br>29. Video game system (attached to TV) (e.g., Xbox, Wii, PlayStation)

**Bedroom media equipment accessibility and availability summary score**	Each media equipment item in the primary child's bedroom was multiplied by its accessibility rating (A=1, B=2, C=3, D=4). The media equipment values in the child's bedroom were then summed.	30. **ACTIVE** video game system (e.g., Wii Fit, Xbox Kinect, PlayStation Move)<br>**Portable**<br>31. Handheld video game player (e.g., Nintendo DS, Sony PSP)<br>32. Laptop computer<br>33. Tablet computer (e.g., iPad, Samsung Galaxy)<br>34. Ipod Touch/ Galaxy Player (or similar)
**Seated furniture accessibility and availability summary score**	Each seated furniture item was multiplied by its accessibility rating (A=1, B=2, C=3, D=4). The seated furniture item values for the home were then summed.	35. Sofa (2+ seater)<br>36. Lounge chair (single seater)<br>37. Coffee table<br>38. Dining/kitchen chair<br>39. Dining/kitchen table<br>40. Office chair<br>41. Desk
**Number of living areas with a TV**	Total number of living areas in the home with a TV	**Living areas**<br>Open plan living area<br>Lounge<br>Office<br>Other room
**Presence of a TV in the child's bedroom**	Whether there was a TV located in the primary child's bedroom	Yes/no
**Presence of an open plan living area in the home**	Whether there was an open plan living area present	Yes/no
Audit questions	***Individual items***	***Item categories***
**Home features**	Type of home	Detached house; Semi-detached; Terrace house; Bungalow; Flat/unit/apartment (5)
	Number of floors	One; Two; More than two (3)
	House size	Small; Medium; Large (3)
	Garden size	Small; Medium; Large; No garden (4)
**Electronic media**	Type of TV service	Freeview; Digital TV (e.g., SKY, Virgin Media, TalkTalk, BT etc…); Other (3)
	Subscription to a movie/TV streaming service? (e.g., Netflix, Now TV, Amazon Video, Kodi etc…)	Yes; No (2)
	Number of smartphones	0; 1-2;3-4;5-6;7-8;>8 (6)
**Space to play**	There is enough space to play…:<br>... in the front garden<br>… in the back garden	Strongly disagree; disagree; agree; strongly agree; (N/A) (5)

	... inside the house	

PA: Physical activity
